# Antioxidant Activity and Anticarcinogenic Effect of Extracts from *Bouvardia ternifolia* (Cav.) Schltdl.

**DOI:** 10.3390/life13122319

**Published:** 2023-12-10

**Authors:** Carmen Valadez-Vega, Olivia Lugo-Magaña, Lorenzo Mendoza-Guzmán, José Roberto Villagómez-Ibarra, Raul Velasco-Azorsa, Mirandeli Bautista, Gabriel Betanzos-Cabrera, José A. Morales-González, Eduardo Osiris Madrigal-Santillán

**Affiliations:** 1 Área Académica de Medicina, Instituto de Ciencias de la Salud, Universidad Autónoma del Estado de Hidalgo, Ex-Hacienda de la Concepción, Tilcuautla, San Agustín Tlaxiaca 42080, Mexico; lencho_1095@hotmail.com; 2 Preparatoria Número 1, Universidad Autónoma del Estado de Hidalgo, Av. Benito Juárez S/N, Constitución, Pachuca de Soto 42060, Mexico; 3 Área Académica de Química, Instituto de Ciencias Básicas e Ingeniería, Universidad Autónoma del Estado del Hidalgo, Ciudad del Conocimiento, Mineral de la Reforma 42184, Mexico; jrvi@uaeh.edu.mx; 4 Área Académica de Biología, Instituto de Ciencias Básicas e Ingeniería, Universidad Autónoma del Estado del Hidalgo, Ciudad del Conocimiento, Mineral de la Reforma 42184, Mexico; raul_velasco@uaeh.edu.mx; 5 Área Académica de Farmacia, Instituto de Ciencias de la Salud, Universidad Autónoma del Estado de Hidalgo, Ex-Hacienda de la Concepción, Tilcuautla, San Agustín Tlaxiaca 42080, Mexico; mibautista@uaeh.edu.mx; 6 Área Académica de Nutrición, Instituto de Ciencias de la Salud, Universidad Autónoma del Estado de Hidalgo, Ex-Hacienda de la Concepción, Tilcuautla, San Agustín Tlaxiaca 42080, Mexico; gbetanzo@uaeh.edu.mx; 7 Laboratorio de Medicina de Conservación, Escuela Superior de Medicina, Instituto Politécnico Nacional, México, Plan de San Luis y Díaz Mirón, Col. Casco de Santo Tomás, Del. Miguel Hidalgo, Ciudad de México 11340, Mexico; jmorales101@yahoo.com.mx (J.A.M.-G.); eomsmx@yahoo.com.mx (E.O.M.-S.)

**Keywords:** antioxidant, *Bouvardia ternifolia*, anticarcinogenic, bioactive

## Abstract

According to the available ethnobotanical data, the *Bouvardia ternifolia* plant has long been used in Mexican traditional medicine to relieve the symptoms of inflammation. In the present study, the cytotoxic effect of extracts obtained from the flowers, leaves and stems of *B. ternifolia* using hexane, ethyl acetate (AcOEt) and methanol (MeOH) was evaluated by applying them to the SiHa and MDA-MB-231 cancer cell lines. An MTT reduction assay was carried out along with = biological activity assessments, and the content of total phenols, tannins, anthocyanins, betalains and saponins was quantified. According to the obtained results, nine extracts exhibited a cytotoxic effect against both the SiHa and MDA lines. The highest cytotoxicity was measured for leaves treated with the AcOEt (ID_50_ of 75 µg/mL was obtained for MDA and 58.75 µg/mL for SiHa) as well as inhibition on ABTS•^+^ against DPPH• radical, while MeOH treatment of stems and AcOEt of flowers yielded the most significant antioxidant capacity (90.29% and 90.11% ABTS•^+^ radical trapping). Moreover, the highest phenolic compound content was measured in the stems (134.971 ± 0.294 mg EAG/g), while tannins were more abundant in the leaves (257.646 mg eq cat/g) and saponins were most prevalent in the flowers (20 ± 0 HU/mg). Screening tests indicated the presence of flavonoids, steroids, terpenes and coumarins, as well as ursolic acid, in all the studied extracts. These results demonstrate the biological potential of *B. ternifolia.*

## 1. Introduction

In Mexico, traditional medicine is still widely used, and such ancestral knowledge of medicinal plants is passed from one generation to another. Although different plant parts are used, depending on the species and the target ailment, leaves and flowers predominate in the traditional recipes, some of which also require the stem or root. Throughout history, herbal remedies have been employed to alleviate symptoms and enhance human health. Even today, in numerous regions worldwide, herbal therapy remains the primary, and at times the sole, option [1].

Medicinal plants are consumed directly or can be prepared as infusions or homeopathic remedies either as a complement to Western medicine or as a stand-alone treatment. While medicinal plants have long been used by indigenous peoples across the globe, their value is increasingly being recognized by the medical profession. Accordingly, evidence of their effectiveness in treating skin conditions, hair loss, herpes, scabies, toothaches and headaches, as well as diseases that weaken the circulatory, digestive, endocrine, nervous, reproductive, respiratory and urinary systems, as well as diseases of cultural affiliation, has grown considerably in recent decades [2,3].

In this context, the *Rubiaceae* family is particularly relevant, as it comprises approximately 650 genera and more than 13,500 species distributed throughout the world, many of which are used in traditional medicine to alleviate headaches and pain during childbirth, as well as lessen the symptoms of autoimmune diseases [4]. In Mexican traditional medicine, the *Bouvardia* genus features most prominently, owing to its anti-inflammatory properties. It can be either ingested or applied as an infusion or compress, to treat intoxication, colic, diarrhea, erysipelas and insect bites, among other conditions [2,5,6]. The firecracker bush or scarlet bouvardia, trumpet bush or clove bush is a 0.5 to 1.5 m. shrub with lustrous, oval, dark green leaves and tubular, bugle-shaped, red flowers, 5 cm long, with the edge flared into four segments. The flowers are arranged in clusters at the ends of numerous erect branches [7].

Consequently, this genus has been subjected to a significant number of phytochemical studies, the findings of which confirm the presence of peptide compounds, such as bouvardin, as well as deoxy-bouvardin and its methylated derivatives, in different plant components [8]. Some secondary metabolites, such as ursolic acid (triterpene) (UA), have also been reported, and flavonoids (such as rutin, quercetin and kaempferol) have been isolated from the aerial part [9,10] of the root, where triterpenes, oleanolic acid (OA) and ursolic acid are also found in high concentrations [11].

The available data also indicate that *B. ternifolia* extracts and different compounds isolated from the plant exhibit cytotoxic activity against some cell lines, causing cell cycle arrest and the inhibition of protein synthesis. In traditional *B. ternifolia* medicinal practices, the plant’s upper parts, encompassing the leaves, stems and flowers, are employed to address conditions such as genital ulcers, dysentery, rabies, cold sweat pains, tumors, fever, and joint pain, and are utilized as a fortifying agent. In addition, they have sedative, analgesic and antispasmodic properties, and are applied for the treatment of snake, bee, scorpion and spider bites [12]. 

Their cytotoxic effect on some malignant cell lines has also been demonstrated, along with the enzymatic inhibition of acetylcholinesterase and anti-inflammatory, analgesic, sedative and hepatoprotective activities. The evidence yielded by in vivo studies further indicates that the *B. ternifolia* extracts can reduce inflammation of the pinna, as well as impart a nootropic effect, thus protecting the nervous system [8,9,13,14]. The aim of the current study was to analyze their antioxidant activity and anticarcinogenic effects on breast cancer cell lines (MDA-MB-231) and cervical cancer cell lines (SiHa). Extracts of the stems, leaves, and flowers of the plant *Bouvardia ternifolia* (Cav.) Schltdl. were evaluated using three organic solvents.

## 2. Materials and Methods

### 2.1. Plant Material 

The plant material required for the current study was collected in the town Epazoyucan in Hidalgo State (north latitude 20°, 01′ and 05″, west longitude 98°, 08′ and 03″) and all specimens were placed on absorbent paper for 20 days to dry at room temperature (20–26 °C). The plant was submitted for identification to the herbarium of the Autonomous University of the State of Hidalgo for classification under the accession number 010. Next, the plant parts (flowers, leaves and stems) were separated and were ground in an electric grinder (analytical mill, 4301-00, Cole Palmer, Vernon Hills, IL, USA) until the powder was sufficiently fine to pass through a 40-mesh. The samples were stored in bags to be preserved until their utilization.

### 2.2. Extract Preparation

The required extracts were obtained from 148 g of flowers, 1450 g of leaves and 1.28 g of ground stems via maceration for 15 days at room temperature in darkness with hexane, ethyl acetate (AcOEt) and methanol (MeOH), followed by filtering on Whatman number 2 paper. The resulting samples were rotaevaporated to dryness (BÜCHI Walter Bath B-480) at 40 °C (330, 240 and 337 mBar), after which the extracts were stored in opaque vials at room temperature until use.

### 2.3. Antioxidant Capacity

ABTS•+ assay: The ABTS•+ assay was performed according to the methodology described in the extant literature [15], using 2,2′ azinobis-(3-ethylbenzothiazoline)-6-sulfonic acid (ABTS•+, Sigma Chemical Co., St Louis, MO, USA) and 6-hydroxy-2, 5, 7, 8-tetramethylcroo-2-carboxylic acid (Trolox, Sigma Chemical Co., St Louis, MO, USA) as the standards. The extracts from *B. ternifolia* were prepared at 100 mg/mL in ethanol. To determine the Trolox concentration (TEAC), 900 µL of the ABTS•+ solution was added to 100 µL of the extract and was reacted for 5 min in darkness, after which the absorbance was measured in a BioTek Epoch spectrophotometer at λ = 734 nm. The results were reported as the percent entrapment and Trolox equivalents in mg/g of the sample (TEAC mg/g) using Trolox as the standard.

DPPH assay: For the DPPH assay, the method developed by Schenk and Brown [16] based on the reduction of the 2,2-diphenyl-1-picrylhydrazyl radical (DPPH• Sigma Chemical Co., St Louis, MO, USA) was used. The extracts were prepared in ethanol at 100 mg/mL, whereby 900 µL of DPPH• solution was added to 100 µL of each extract and the sample was left to react in darkness for 60 min before reading the absorbance at λ = 734 nm (BioTek Epoch instrument, Santa Clara, CA, USA). The results were reported as the Trolox equivalent antioxidant capacity in mg/g of the sample (TEAC mg/g) using a calibration curve with Trolox as the standard.

### 2.4. Phytochemical Analysis

Total phenol content: For total phenol determination, the spectrophotometric method described by Singleton [17] was employed. Extracts from *B. ternifolia* were used. To determine the total phenolic content, 100 µL of a 1 mg/mL sample from each extract, 500 µL of Folin’s solution and 400 µL of Na_2_CO_3_ were added. The reaction mixture was allowed to react in darkness for 30 min and then measured at a wavelength of 765 nm using a microplate reader (BioTek Epoch instrument). The results were reported in mg gallic acid equivalents per gram of sample (mg EGA/g sample), using gallic acid as the standard (Sigma Chemical Co., St Louis, MO, USA).

Tannin content: The extracts were prepared in methanol at a concentration of 4 mg/mL, by shaking for 1 h. Then, 0.5 mL of the extract was mixed with 2.5 mL of a 0.5% vanillin solution (Sigma Chemical Co., St. Louis, MO, USA). It was reacted for 45 min at room temperature and the absorbance was measured at λ = 500 nm (BioTek Epoch instrument). A calibration curve was performed using (+) catechin (Sigma Chemical Co., St. Louis, MO, USA) as a standard and the results were expressed as mg CATE/g (milligram catechin equivalents per gram of sample) [18]. 

Anthocyanin content: For determining the anthocyanin content, organic extracts (0.3 mg/mL) were left to react in acidified ethanol (0.2%) overnight in darkness, after which the samples were filtered and diluted in ethanol-HCl at 4 °C. The total content of monomeric anthocyanins was quantified using the differential pH method (109), for which extract solutions were prepared at a pH of 1 (KCl 0.1 M) and a pH of 4.5 (CH_3_COOH/CH_3_COO-). Finally, the absorbance was measured in the λ = 515–700 nm wavelength range (BioTek Epoch instrument) [19].

Betalain content: The betalain content was determined using the spectrophotometric method described by Elbe [20]. For this purpose, betalains were obtained from each *B. ternifolia* extract in phosphate buffer at a pH if 6.5 (17 mg/mL), after which the samples were vortexed and centrifuged at 5000 rpm for 15 min at 4 °C. Next, the supernatant was filtered in Phenomenex 0.45 µm, and the absorbance was measured at λ = 538 and 483 nm (Epoch-BioTek Instrument). The betacyanin and betaxanthin content was quantified using the method described in the extant literature [21]. The betacyanin content was expressed as mg betanin/100 g sample (mg BE/100 g), and the betaxanthin content was expressed as mg vulgaxanthin-I/100 g sample (mg VE/100 g) [21].

Saponin content: To determine the saponin content, the extracts were subjected to the methodology described by Valadez, extracted for 1 h from 10 mg of the extract sample using 85:15 (%) methanol–water solution. The solvents were removed via evaporation, and the extracted saponins were diluted in a NaCl solution. Employing a serial dilution method with human erythrocytes type O in a U-shaped microtiter 96-well plate, the saponin-containing solution underwent a 2-fold serial dilution. The volume of each sample in the wells was adjusted to 50 μL with NaCl (0.9%), and the resulting diluted samples were combined with 50 μL of a 4% erythrocyte suspension. The reaction mixture underwent a 1 h incubation at room temperature, and the maximum dilution demonstrating hemolysis was subsequently observed. The analyses were conducted in triplicate, and the results were quantified and reported as hemolytic units [22].

### 2.5. Cytotoxicity Assay

The assay was performed as described by Valadez-Vega [23]. The MDA-MB-231 (human breast adenocarcinoma) and SiHa (cervix squamous cancer cells) cell lines used for this purpose were obtained from the American Type Culture Collection (ATCC, Rockville, MD, USA). The cells were propagated in Dubelco’s Modified Eagle Medium (DMEM) supplemented with 10% fetal bovine serum (FBS) and 0.1% antibiotic (a combination of streptomycin and penicillin). Incubation was carried out at 37 °C in a humidified atmosphere with 5% CO_2_. Passages between 10 to 25 were routinely employed for both cell lines [24].

The analysis was performed using the colorimetric method described by Mosmann, focusing on the mitochondrial functionality of the treated cells [25]. MTT assay is a colorimetric test that utilizes a tetrazolium dye known as 3-(4,5-dimethylthiazol-2-yl)-2-5-diphenyltetrazolium bromide (MTT) to assess the viability of cell lines. This assay relies on the capacity of active mitochondria to facilitate the conversion of MTT into a solid formazan, the amount of which can be quantified via spectrophotometric means. Prior to the measurements, both cell lines were cultured in 96-well microplates (1 × 10^4^ cell/well) in a culture medium containing fetal bovine serum and antibiotics. After 24 h incubation, the culture medium was removed and was replaced by the extracts in 0–2000 µg/mL concentration. Following further 24 h incubation, the solution containing the cells was replaced with MTT (5 mg/mL) and was incubated at 37 °C for 3 h. Then, the culture medium was removed and 200 µL of dimethyl sulfoxide was added to each well to dissolve the formazan compound produced by the cells. The absorbance of each well was measured at λ = 540 nm (BioTek Epoch instrument) and the cell viability percentage was calculated considering the blank as 100% viability.

### 2.6. Statistical Analyses

An ANOVA and Tukey’s test (*p* < 0.05) were conducted to determine the differences between extracts in terms of the antioxidant capacity, scavenging capacity, phenol content, tannin content and saponin content. All statistical analyses were performed using StatGraphics Centurion version 19.1 (StatGraphics, The Plains, VA, USA). 

## 3. Results

### 3.1. Antioxidant Capacity

**Figure 1:**
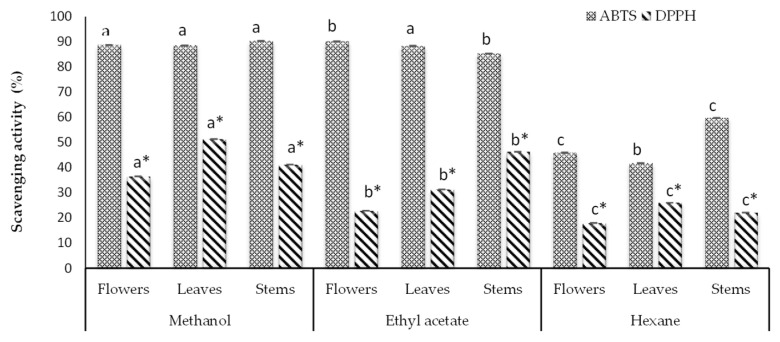
Scavenging capacity of *B. ternifolia* extracts based on the findings yielded by ABTS•^+^ and DPPH• assays based on 6.66 mg/mL extract concentration. The statistically significant differences (*p* < 0.05) in values obtained from different plant parts based on the ABTS•^+^ and DPPH• assay are denoted with a, b and c, and a*, b*and c*, respectively. The average of three independent assays ± standard deviation is shown.

The antioxidant capacity of the studied *B. ternifolia* extracts from flowers, leaves and stems was evaluated by applying the ABTS•+ and DPPH• assays. The obtained findings are depicted in Figure 1 and reported in Table 1, revealing significant differences between these two approaches with respect to all three morphological parts (*p* < 0.005) with the ABTS•+ assay consistently yielding greater values. 

### 3.2. Total Phenol, Tannin and Saponin Content

The total phenol, tannin and saponin content in the extracts obtained from the *B. ternifolia* flowers, leaves and stems was determined using methanol, ethyl acetate and hexane as solvents, and the findings are reported in Table 2.

### 3.3. Cytotoxicity of the Extracts Obtained from the B. ternifolia Flowers, Leaves and Stems against MDA and SiHa Cells

**Figure 2:**
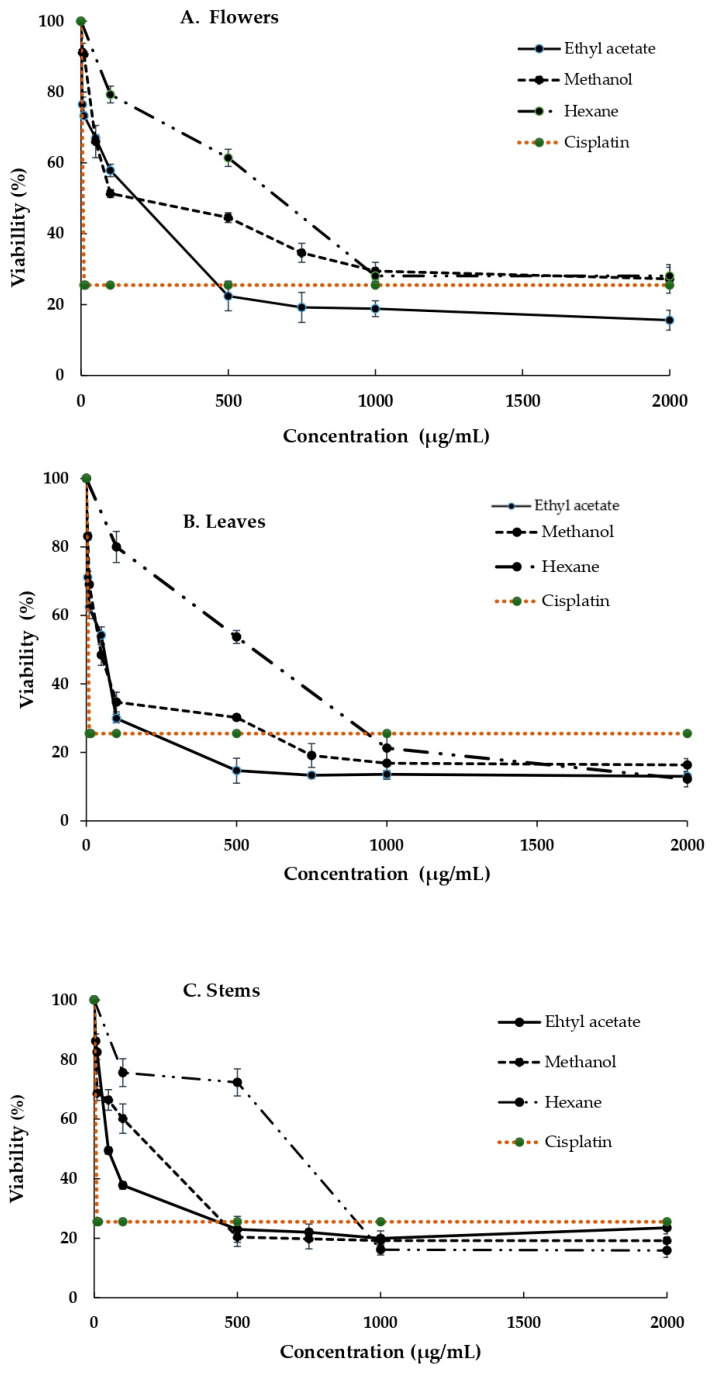
Cytotoxic effect of *B. ternifolia* extracts on the SiHa cell line (**A**) Flowers, (**B**) Leaves and (**C**) stems in three different solvents. Cells were exposed for 24 h to different concentrations of extracts, and, after incubation, cell viability was measured using the MTT technique. All values are expressed as the mean ± standard deviation of three independent experiments.

**Figure 3:**
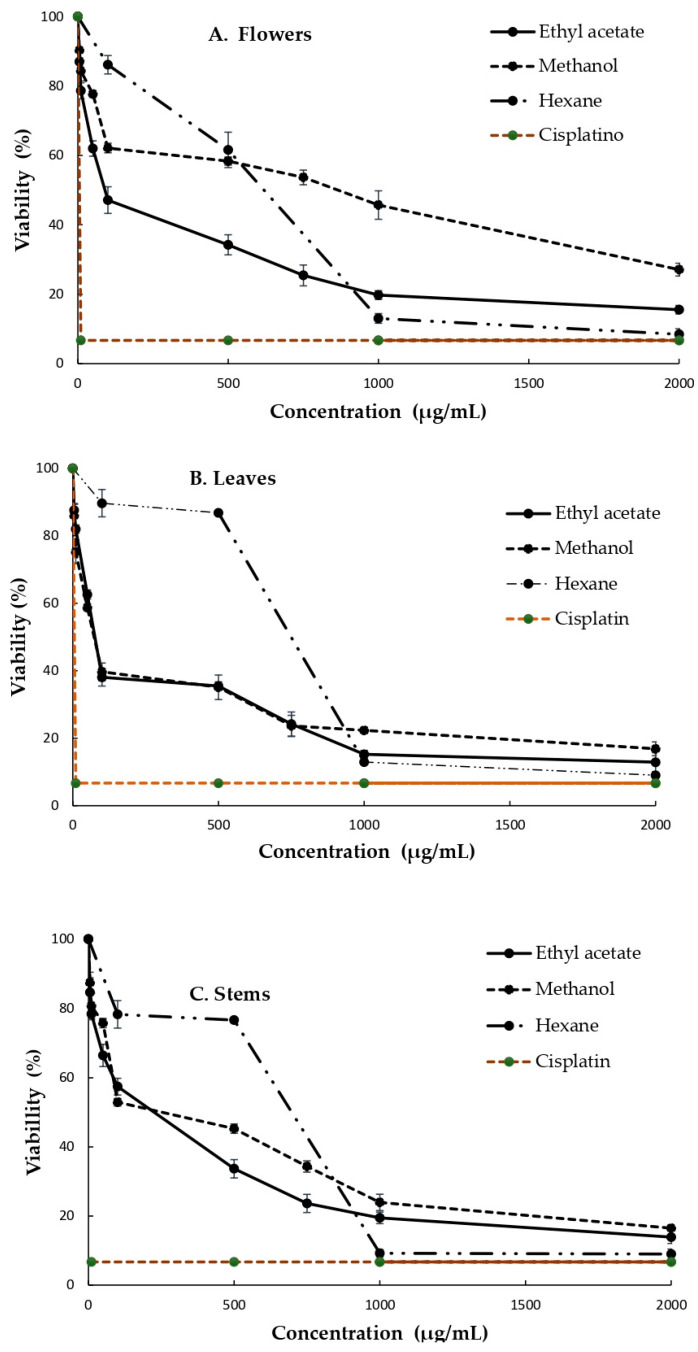
Cytotoxic effect of *B. ternifolia* extracts on the MDA cell line (**A**) Flowers, (**B**) Leaves and (**C**) stems in three different solvents. Cells were exposed for 24 h to different concentrations of extracts, and, after incubation, cell viability was measured using the MTT technique. All values are expressed as the mean ± standard deviation of three independent experiments.

As shown in Figure 2 and Figure 3, the extracts have a cytotoxic effect on the MDA and SiHa cell lines, which is more pronounced in the SiHa case. As can be seen from the tabulated results, while all extracts had a dose-dependent effect, the extracts from the leaves and stems obtained using MeOH and AcOEt exhibited the highest cytotoxicity on the SiHa cell line, while the lowest cytotoxicity was measured in the extracts from flowers and stems obtained using hexane.

It can also be concluded from Figure 2A that the flowers extract in ethyl acetate had the greatest cytotoxic effect on the SiHa cell line, while the leaf extract obtained using AcOEt exhibited the highest inhibition of cell viability (Figure 2B) and the methanolic stem extract showed the greatest effect on this cell line (Figure 2C).

The corresponding findings related to the MDA cell line are shown in Figure 3, revealing that the flower extract obtained with ethyl acetate has the greatest cytotoxic effect on the SiHa cell line (Figure 3A), while the AcOEt- and MeOH-based extracts from the leaves were the most effective in reducing cell viability (Figure 3B). On the other hand, the hexane extract caused the greatest inhibition of cell viability at high concentrations (Figure 3C).

When the same tests were performed with the MDA cell line, the ethyl acetate extract presented the greatest inhibition of cell viability irrespective of the concentration used.

## 4. Discussion

The results obtained in the present study indicate that the ABTS•^+^ method shows a greater scavenging capacity than DPPH• for the MeOH, AcOEt and hexane extracts from different parts of the *B. ternifolia* plant. The ABTS•^+^ assay produced greater values when applied to the extracts: the ABTS•^+^ method was considered to show higher sensitivity when applied to plant extracts containing hydrophilic, lipophilic and highly pigmented antioxidant compounds compared to the DPPH method [26].

Mosquera, focusing on species belonging to the families *Euphorbiaceae* and *Asteraceae*, found significantly higher scavenging percentages when MeOH extracts were tested in DPPH• above 50%; similar results were found in this investigation (22.13–51.32%), where a value of more than 25% is considered indicative of active antioxidants [27]. In general, highly pigmented and hydrophilic antioxidants, such as those at the focus of the current investigation, tend to have a better response in ABTS•^+^ assays than in DPPH• [28], suggesting the presence of phenolic acids, flavonoids and other polyphenols that are closely related to the antioxidant capacity [29,30]. Still, it is worth noting that the DPPH• technique is more selective, as ABTS•^+^ does not react with flavonoids that lack hydroxyl groups in the B-ring, or with aromatic acids that contain a single hydroxyl group [31]. Irrespective of these differences, in the present study, the antioxidant capacity and the content of phenolic compounds obtained using both methods correlated with the content of total phenolic compounds [32].

When the ABTS•^+^ assay was adopted, the greatest amount of total phenols in the AcOEt extracts from the *B. ternifolia* stems and MeOH from the leaves was measured, along with the highest antioxidant activity, supporting the previous reports related to the extracts of the same polarity of *Palicourea guianensis* [33]. As expected, AcOEt extracts from *B. ternifolia* flowers and hexane from the stems presented a lower phenol content and antioxidant capacity, as the total phenol content is closely related to the antioxidant activity [34]. The chemical complexity of plant extracts makes it difficult to explain and interpret antioxidant activity.

Due to the complexity of the oxidation–antioxidation processes, it is obvious that no single test method is able to provide a complete picture of the antioxidant profile of a sample under study [35].

The ABTS•^+^ establishing the relationship between the total phenol content and antioxidant activity was compared to DPPH•. As noted by Javanmardi et al. [36], the antioxidant capacity of phenolic compounds mainly stems from their redox properties, which play an important role in neutralizing free radicals at the cellular level. Accordingly, in several members of the *Rubiaceae* family—such as *Palicourea petiolaris wemh*, *Palicourea andaluciana* and *Palicourea thyrsiglora*—antioxidant activity has already been confirmed [27]. Given the intricate nature of plant extracts, elucidating antioxidant activity remains a complex task. It is evident that no single test method can comprehensively depict the complete antioxidant profile of a given sample. The ABTS•+ assay proves to be particularly valuable in establishing the relationship between total phenol content and antioxidant activity, surpassing the capabilities of the DPPH• method. This underscores the significance of the redox properties of phenolic compounds in neutralizing free radicals at the cellular level, as evidenced by previous studies in certain members of the *Rubiaceae* family. It is worth noting that, as of now, there are no existing reports on the total phenols in species of the genus *Bouvardia.*

In the present study, the MeOH and AcOEt leaf extracts contained a higher amount of tannins, 134.71 and 257.64 mg CATE/g, respectively. These values are lower than those reported for *Simira mexicana* and *Randia echinocarpa* leaf extracts, namely 7.58 and 1.06 mg CATE/g, respectively. However, phenol content is not indicative of tannin production, as phenols can give rise to other compounds such as phenolic acids, stilbenes, lignans, phenolic alcohols and flavonoids [37,38].

Tannins are found in all organs or parts of the plant (stems, wood, leaves, seeds and domes) and can contribute to 2–7% of the fresh weight of the plant, depending on the species, climate, soil, temperature and other factors [38].

In the present study, when the saponin assay was employed, only the MeOH extract obtained from flowers showed hemolytic activity at 20 HU/mg, which was expected as saponins are amphipathic and glycosidic in nature. Accordingly, they can be found in solvents of higher polarity [39,40]. Previously, Giraldo and Ramírez reported that extracts with higher polarity have a higher saponin content for *Palicourea guianensis* (Rubiaceae) leaves, supporting the findings reported in this work [33]. On the other hand, in the 250–370 nm and 500–545 nm wavelength ranges, the anthocyanin quantification method failed to provide any evidence of these compounds [41].

Several factors can influence anthocyanin degradation, including temperature, light, solvents, storage time and pH [21]. Therefore, the absence or low amount of betalains in the studied extracts is expected given that these compounds have only been observed in families of the order Caryophyllales, and *Bouvardia* belongs to the order Gentianales [42].

Compounds derived from plants, along with their semisynthetic and synthetic counterparts, constitute a prominent reservoir of pharmaceuticals for treating human diseases. Within the realm of cancer therapy, a key focus lies in identifying plant proteins that exhibit robust cytotoxic activity while maintaining low toxicity, and that exert diverse mechanisms of action on tumors. The cell viability results revealed the greater cytotoxic effect of *B. ternifolia* leaf and stem extracts compared to flower extracts irrespective of the solvent used or the cell line. Still, the MeOH leaf extract had the greatest cytotoxic effect on the MDA-MB-231 cell line, whereas the SiHa cell line was more susceptible to the cytotoxicity of the MeOH and AcOEt leaf extracts. On the other hand, the AcOEt extracts from the flowers and leaves and MeOH extract from the stems were the most inhibitory to the viability of the SiHa cell line. 

Currently, no cytotoxicity studies on *B. ternofolia* have been reported; however, Rupachandra and Sarada studied= the seeds of *Borreria hispida*, a member of the Rubiaceae family, which revealed that the seeds have cytotoxic activity against lung (A549) and cervical (HeLa) cancer cell lines [43]. 

Within the Rubaceae family, Thai noni/Yor (*Morinda citrifolia* Linn.) were extracted using several methods and evaluated against human cancer cell lines: KB (human epidermoid carcinoma), HeLa (human cervical carcinoma), MCF-7 (human breast carcinoma) and HepG2 (human hepatocellular carcinoma) cell lines, as well as a Vero (African green monkey kidney) cell line, employing the MTT colorimetric method, and the dichloromethane extract of the leaves had a higher inhibitory effect on the HeLa cells [44]. 

However, as the cytotoxic activity of *B. ternifolia* on the cervical carcinoma cell line SiHa has never been reported, direct comparisons of the results obtained in this work are not possible.

In the MDA-MB-231 cells, the greatest inhibition of viability was achieved by the hexane extracts from flowers and stems and AcOEt extracts obtained from leaves. In other studies, the cytotoxic activity of *Chiococca alba*, *Hamelia patens* extracts showed no cytotoxic activity in Hep-G2 and MDA-MB-231 cells, while *Posokeria latifolia* extracts yielded 10.56% inhibition [45]. The ID_50_ of AcOEt and MeOH leaf extracts (with the values of 75.66 and 72.87 µg/mL, respectively) reflected higher cytotoxic activity. Similar results were observed for compounds isolated from the branches and leaves of *Heinsa crinita*, indicating cytotoxic activity in HL-60, SMMC-7721, A-549, MCF-7 and SW-480 cells, with ID_50_ in the 3.11–20.12 µg/mL range [46].

MeOH extracts from the leaves and branches of *Pavetta indica L.* were shown by Nguyen et al. to exhibit a cytotoxic effect on MDA-MB-231 cells, whereby ID_50_ = 25.2 µg/mL was measured at 24 h. Likewise, extracts from the bark of *Hymenodictyon excelsum*, belonging to the Rubiaceae family, had significant cytotoxic effects on both MCF-7 and MDA-MB-231 cells, with an ID_50_ of 80 and 440 µg/mL, respectively [47]. 

In previous studies, *Morinda citrifolia* Linn extract in ethyl acetate also showed a cytotoxic effect on MCF-7 and MDA-MB-231 cells with an ID_50_ of 25 and 35 µg/mL, as well as increased apoptosis in these cell lines, while it arrested the cell cycle in the G1/S phase in MCF-7 and G0/G1 in MDA-MB-231 -MB-231 [43]. According to the prior analyses of bouvardin (BVB), deoxybouvardin and its methylated derivatives, which have already been identified and isolated from *B. ternifolia*, are capable of inducing the proliferation of Chinese hamster ovary (CHO) cells, in which BVB reduced the ability to pass through the cell cycle [13]. Furthermore, BVB is effective in inhibiting protein synthesis in leukemia (P388) and melanoma (B16) cells [48]. 

For future investigations, it is proposed to explore the specific bioactive compounds within the extracts demonstrating cytotoxic effects, employing techniques such as chromatography and spectroscopy to isolate and identify these compounds. Additionally, mechanistic studies on cytotoxicity should be conducted to elucidate the pathways through which the identified compounds exert cytotoxic effects on SiHa and MDA-MB-231 cancer cell lines. This may involve the application of molecular biology techniques to investigate phenomena like cell cycle arrest, apoptosis, or autophagy induction. Finally, the transition from in vitro studies to in vivo experiments is needed to validate the observed cytotoxic effects in cell lines. Animal models must be utilized to assess the safety, bioavailability and efficacy of *B. ternifolia* extracts within a more complex biological context.

## 5. Conclusions

The observed cytotoxic effects of the studied extracts exhibited distinct characteristics in comparison to the findings reported by other researchers, particularly evident in their significant impact on both cell lines. The variability in outcomes could potentially be attributed to differences in the concentration and composition of secondary metabolites present in the *Bouvardia ternifolia* plant. 

The phytochemical analyses conducted revealed the presence of saponins, tannins and phenolic compounds. Notably, extracts from the leaves exhibited the highest concentrations of tannins and phenols, suggesting a potential influential role in cellular viability.

It is noteworthy that this study represents the inaugural exploration utilizing SiHa and MDA-MB-231 cell lines to assess the cytotoxic effects of *Bouvardia ternifolia*, thus contributing novel insights to Mexican traditional medicine. Furthermore, this research lays the groundwork for future investigations, both biological and phytochemical, aimed at comprehensively understanding the therapeutic potential and chemical constituents of this plant species.

## Figures and Tables

**Table 1:** Antioxidant capacity of *B. ternifolia* extracts.

Solvent	Parts of Plants	Antioxidant Capacity	
		DPPH•	ABTS•+
		mg TEAC/g	
Methanol	Flowers	16.909 ± 0.040 ^a^	22.166 ± 0.043 ^a^
	Leaves	23.142 ± 0.054 ^a^	22.123 ± 0.043 ^a^
	Stems	18.875 ± 0.054 ^a^	22.551 ± 0.043 ^a^
Ethyl acetate	Flowers	11.168 ± 0.060 ^b^	22.509 ± 0.043 ^a^
	Leaves	14.737 ± 0.040 ^b^	22.08 ± 0.042 ^a^
	Stems	21.013 ± 0.045 ^b^	22.737 ± 0.025 ^a^
Hexane	Flowers	9.134 ± 0.040 ^c^	12.165 ± 0.049 ^b^
	Leaves	12.522 ± 0.015 ^c^	11.194 ± 0.042 ^b^
	Stems	10.866 ± 0.040 ^c^	15.394 ± 0.042 ^b^

ABTS•+ and DPPH• results are expressed as Trolox equivalent antioxidant activity (TEAC). The average of three independent replicates ± standard deviation is reported and the a, b and c superscripts indicate statistically significant differences (*p* < 0.05) across different plants depending on the solvent used.

**Table 2:** The total phenol, tannin and saponin content in the extracts obtained from the *B. ternifolia* flowers, leaves and stems.

Parts of Plant	Solvent	Phenols (mg GAE/g)	Tannins (mg CATE/g)	Saponins (HU/mg)
	Methanol	78.794 ± 0.294 ^a^	43.721 ± 5.747 ^b^	20
Flowers	Ethyl acetate	37.618 ± 0.304 ^b^	34.143 ± 4.619 ^a^	ND
	Hexane	51.147 ± 0.284 ^c^	37.974 ± 1.437 ^ab^	ND
	Methanol	133.402 ± 0.34 ^a^	134.717 ± 6.789 ^a^	ND
Leaves	Ethyl acetate	71.441 ± 0.294 ^b^	257.646 ± 27.201 ^b^	ND
	Hexane	43.696 ± 0.170 ^c^	32.706 ± 2.991 ^c^	ND
	Methanol	117.324 ± 0.29 ^a^	58.089 ± 2.488 ^a^	ND
Stems	Ethyl acetate	134.971 ± 0.89 ^b^	61.920 ± 3.616 ^a^	ND
	Hexane	8.794 ± 0.09 ^c^	32.706 ± 5.982 ^b^	ND

Total phenols are expressed in gallic acid equivalents (GAE), tannins are expressed in catechin equivalents (CATE) and saponins are presented in hemolytic units per milligram (HU/mg). ND: Not detected. The average of three independent replicates ± standard deviation is reported. The a, b and c superscripts indicate statistically significant differences (*p* < 0.05) across different plants depending on the solvent used.

## Data Availability

The data presented in this study are available in this article.
